# Comprehensive Profiling of Essential Elements and Organic and Inorganic Contaminants in Dromedary Camels from the Canary Islands: A Baseline for Nutritional and Environmental Assessment

**DOI:** 10.3390/vetsci12090829

**Published:** 2025-08-29

**Authors:** Andrea Acosta-Dacal, Adrián Melián Henríquez, Juan Alberto Corbera, Ana Macías-Montes, Manuel Zumbado, Norberto Ruiz-Suárez, José Luis Martín-Barrasa, Octavio P. Luzardo, María Teresa Tejedor-Junco

**Affiliations:** 1 Research Institute of Biomedical and Health Sciences (IUIBS), Universidad de Las Palmas de Gran Canaria, Paseo Blas Cabrera Felipe “Físico” 17, 35016 Las Palmas de Gran Canaria, Spain; andrea.acosta@ulpgc.es (A.A.-D.); adrian.melian@ulpgc.es (A.M.H.); juan.corbera@ulpgc.es (J.A.C.); ana.macias@ulpgc.es (A.M.-M.); manuel.zumbado@ulpgc.es (M.Z.); norberto.ruiz@ulpgc.es (N.R.-S.); octavio.perez@ulpgc.es (O.P.L.); 2 Hospital Clínico Veterinario, Universidad de Las Palmas de Gran Canaria (HCV-ULPGC), Campus Universitario de Arucas, 35413 Arucas, Spain; 3 Spanish Biomedical Research Centre in Physiopathology of Obesity and Nutrition (CIBERObn), 28029 Madrid, Spain; 4 Research Unit of the Hospital Universitario of Gran Canaria Dr. Negrín, Fundación Canaria del Instituto de Investigación Sanitaria de Canarias (FIISC), C. Pl. Barranco de la Ballena s/n, 35019 Las Palmas de Gran Canaria, Spain; joseluis.martin@ulpgc.es; 5 IUSA-ONE HEALTH 2—Sanidad Animal de la Acuicultura y Especies Silvestres, Enfermedades Infecciosas y Seguridad Alimentaria, University Institute of Animal Health and Food Safety (IUSA), University of Las Palmas de Gran Canaria, 35416 Arucas, Spain; 6 Eukaryotic-Prokaryotic Synergy—Comparative and Translational Medicine (Cardiorespiratory Infectious Diseases and Epidemiology Group), Fundación Canaria del Instituto de Investigación Sanitaria de Canarias (FIISC), 35012 Las Palmas de Gran Canaria, Spain; 7 CIBER de Enfermedades Infecciosas (CIBERINFEC), Instituto de Salud Carlos III, 28029 Madrid, Spain

**Keywords:** serum biomonitoring, One Health, veterinary public health, ICP-MS, UHPLC-MS/MS, trace elements, rare earth elements, semi-extensive farming

## Abstract

Dromedary camels raised under semi-extensive management can act as One Health sentinels for environmental exposures and food chain surveillance, yet serum reference information remains scarce. Our objective was to provide the most comprehensive assessment to date of physiological and toxicological serum profiles in dromedary camels (*Camelus dromedarius*) from the Canary Islands. We included 114 clinically healthy animals of different sex, age, and reproductive status. Serum samples were analyzed for essential, toxic, and potentially toxic elements using inductively coupled plasma mass spectrometry (ICP-MS). In addition, a high-throughput multi-residue method based on QuEChERS extraction followed by UHPLC-MS/MS and GC-MS/MS was used to screen for 360 organic compounds, including pesticides, veterinary drugs, human pharmaceuticals, and persistent organic pollutants. Essential elements showed biologically consistent variations according to sex, age group, and pregnancy status. Males had higher levels of selenium and copper, while calves showed elevated concentrations of manganese and zinc. Pregnant females exhibited lower iron, zinc, and selenium levels, consistent with increased fetal demand. These results provide preliminary reference values for healthy camels, stratified by physiological status. In contrast, classical toxic elements such as arsenic, mercury, lead, and cadmium were found at very low or undetectable concentrations. Several potentially toxic elements, including barium, strontium, and rare earth elements, were detected sporadically but without toxicological concern. Only 13 organic compounds (3.6%) were detected in any sample, and concentrations were consistently low. The most prevalent was the PAH acenaphthene (55.3%), followed by the fungicide procymidone and the PAH fluorene. Notably, no residues of the usually detected 4,4′-DDE or PCB congeners were found in any sample. These findings confirm the low environmental and dietary exposure of camels under low-intensity farming systems and highlight their value as sentinel species for food safety and environmental monitoring.

## Simple Summary

Serum biomonitoring is increasingly used to characterize the chemical footprint of husbandry systems with low intensification. Dromedary camels managed under semi-extensive conditions in the Canary Islands offer a unique model linking animal health, food chain surveillance, and the surrounding environment. Here, we provide a broad, field-applicable serum baseline for essential/macroelements and toxic elements, together with a comprehensive multi-residue screening of organic contaminants. Rather than assessing regulatory compliance, our goal is to deliver practical reference information for veterinarians and producers and to support future One Health monitoring in camel herds.

## 1. Introduction

Dromedary camels (*Camelus dromedarius*) have played a multifaceted role in the livelihoods of human societies for centuries. Originally native to arid regions of North Africa and the Middle East, these animals have been essential not only as pack and draft animals but also as sources of milk, meat, wool, and leather [1]. Their remarkable adaptations to harsh climates—including high thermal tolerance, water efficiency, and a capacity to thrive on poor-quality forage—have made them especially relevant in the face of increasing desertification and climate change [2]. In recent years, this ancient species has undergone a process of renewed interest, with dromedary farming gaining ground not only in its traditional strongholds but also in Europe and Asia, spurred by the increasing demand for camel milk and other products considered functional or nutraceuticals [1,2,3,4].

Within the European context, the Canary Islands represent a unique biogeographic and historical enclave where the dromedary has been present for over five centuries. Introduced from North Africa in the 15th century, camels in the archipelago were historically used for agricultural work, transportation, and even military service [5]. Although their economic significance declined with mechanization, these animals have persisted as a cultural and touristic asset and, more recently, have experienced a resurgence in production-oriented settings, particularly for milk and meat [2,3,4,6]. As a result, the Canary Islands provide a rare opportunity to evaluate camel husbandry under semi-extensive, European conditions that differ markedly from the traditional desert-based systems.

Alongside this renewed zootechnical interest, there is growing concern about the safety of products derived from camels, especially given their entry into the human food chain. While camel milk is often marketed as hypoallergenic and rich in antioxidant and antimicrobial peptides [2,3], it is important to evaluate whether these potential benefits are compromised by environmental exposure to chemical pollutants. In fact, studies on food-producing animals in other parts of the world have demonstrated the capacity of livestock to accumulate a wide range of contaminants through their diet, water sources, and environmental surroundings, with possible repercussions for animal health, reproduction, and human exposure through consumption [7,8].

Despite their ecological and productive value, camels have been relatively underrepresented in biomonitoring studies. Research conducted in regions such as Egypt, Mauritania, and Mongolia has highlighted the presence of trace metals [9,10], organochlorine pesticides [11], and persistent organic pollutants (POPs) such as polychlorinated biphenyls (PCBs) in camel tissues. Although these studies are informative, they are often geographically restricted and analytically limited in scope, typically focusing on a small set of compounds and tissues.

In contrast, modern analytical techniques now enable the simultaneous determination of hundreds of substances—both organic and inorganic—across various biological matrices. These high-throughput approaches are particularly relevant for non-traditional livestock such as camels, where limited baseline data exist and where exposure pathways may differ from more intensively managed species. Moreover, the inclusion of a broad panel of analytes—ranging from essential elements (e.g., Se, Zn, Cu) to toxic and potentially toxic metals (e.g., Pb, Cd, Hg), pesticides (e.g., chlorpyrifos, endosulfan), and emerging pollutants—provides a more comprehensive view of the chemical burden borne by these animals and the potential for cumulative or synergistic risks [12].

Particularly concerning are the effects of chronic exposure to trace elements and synthetic compounds on reproductive health. Numerous studies have shown that even low levels of cadmium, lead, mercury, or arsenic can alter spermatogenesis, reduce semen quality, and impair endocrine function in male mammals, including ruminants and pseudoruminants [12]. Meta-analyses and systematic reviews have shown consistent associations between metal exposure and subfertility, highlighting oxidative stress, endocrine disruption, and mitochondrial dysfunction as key mechanistic pathways [12,13,14]. While these effects have been well documented in cattle, sheep, and goats, their extrapolation to camels remains speculative due to the scarcity of species-specific data [13].

In addition to reproductive impacts, chemical exposure in camels can affect immune function, liver and kidney health, and the quality and safety of products derived from these animals [13,15,16]. Previous reports have identified residues of banned pesticides such as DDT, dieldrin, and lindane in camel meat and milk in African and Asian countries, raising concerns about the persistence of legacy contaminants in regions with lax regulatory enforcement [11,15]. In parallel, recent reviews have reported the presence of heavy metals in milk consumed globally, noting that levels of Pb and Cd occasionally exceed regulatory thresholds and may pose health risks, particularly for vulnerable populations, such as children [8].

From a food safety perspective, integrating camels into formal production systems requires rigorous baseline data on contaminant levels and their variability across individuals and environmental conditions. This is especially relevant in insular ecosystems such as the Canary Islands, where exposure pathways may include atmospheric deposition, anthropogenic waste, agricultural runoff, and natural geochemical sources [17,18].

To address these knowledge gaps, we designed a study that combines high-resolution mass spectrometry and inductively coupled plasma techniques to assess the presence and concentrations of over 400 analytes in serum samples from dromedary camels reared under semi-extensive conditions in the Canary Islands. This comprehensive approach enables simultaneous determination of essential elements relevant to the animal’s nutritional and physiological status, as well as toxic and potentially toxic elements and a wide range of organic contaminants—including pesticides, persistent organic pollutants (POPs), and pharmaceuticals. This study aimed to establish serum baselines for physiological (essential/macro) and toxic elements and to screen a comprehensive panel of organic residues in dromedary camels under semi-extensive management to inform veterinary practice and One Health surveillance. By integrating both physiological and toxicological endpoints, this study offers a robust diagnostic framework for environmental monitoring and food safety surveillance. The findings not only contribute baseline reference values for healthy camels across different physiological categories but also support the development of evidence-based guidelines for the safe and sustainable inclusion of camel-derived products in European food systems.

## 2. Materials and Methods

The present study was carried out at the Veterinary Teaching Hospital of the Faculty of Veterinary Medicine, University of Las Palmas de Gran Canaria (ULPGC), as part of the ongoing research program on Dromedary Medicine. Analytical determinations were conducted at the Toxicology Unit of the Research Institute of Biomedical and Health Sciences (IUIBS), ULPGC. All procedures involving animals were reviewed and approved by the Ethics Committee for Animal Experimentation of ULPGC (OEBA-ULPGC—12/2023).

### 2.1. Animals and Sample Collection

A total of 116 clinically healthy dromedary camels (*Camelus dromedarius*) were included in the study. All animals belonged to three mixed agricultural–livestock holdings located in Fuerteventura (Canary Islands, Spain) and were managed under uniform feeding and husbandry. Sampling was performed during routine handling using non-probability convenience sampling: within each herd, consecutive animals meeting the inclusion criteria were selected. Owners provided herd records and consent, and sampling was conducted by the attending veterinarian. For comparisons, calves were defined as <12 months and adults as ≥12 months; age was obtained from herd records. Pregnancy was assigned from farm reproductive records and on-site clinical assessment at sampling (recorded gestation or positive clinical diagnosis = pregnant; otherwise = non-pregnant). Each animal was individually identified by microchip. Date of birth, sex, and reproductive status, including pregnancy when applicable, were recorded at the time of sampling.

Inclusion criteria required that all camels be clinically healthy, as determined by thorough physical examination, with no history of previous illness or pharmacological treatment within the six months preceding sample collection.

Blood samples were obtained by jugular venipuncture into vacuum tubes without anticoagulant (clot activator) to allow clotting and serum separation. After clotting at room temperature, tubes were centrifuged, and the serum was aliquoted and stored at −20 °C until analysis. No whole-blood aliquots in anticoagulant tubes were collected. All samples were immediately placed on ice and transported to the laboratory for processing. Given the year-round climatic stability of the Canary Islands, seasonal variation was not considered a confounding factor in this study.

### 2.2. Inorganic Analysis

In this study, we quantified the concentration of 55 inorganic elements in serum samples from dromedary camels (*Camelus dromedarius*), encompassing a wide array of elements with nutritional, toxicological, and environmental relevance. The analytical panel included physiologically essential trace elements such as lithium (Li), manganese (Mn), iron (Fe), cobalt (Co), zinc (Zn), copper (Cu), selenium (Se), and molybdenum (Mo), as well as macroelements fundamental to cellular and metabolic function, including sodium (Na), magnesium (Mg), phosphorus (P), sulfur (S), potassium (K), and calcium (Ca). We also included four well-known toxic heavy metals—arsenic (As), cadmium (Cd), mercury (Hg), and lead (Pb)—given their well-documented adverse health effects even at low concentrations. A complementary group of potentially toxic or environmentally persistent elements was also analyzed, including beryllium (Be), boron (B), titanium (Ti), vanadium (V), chromium (Cr), nickel (Ni), strontium (Sr), tin (Sn), antimony (Sb), cesium (Cs), barium (Ba), bismuth (Bi), thallium (Tl), thorium (Th), and uranium (U), all of which are relevant for environmental biomonitoring in regions influenced by anthropogenic activities. Finally, we incorporated a comprehensive panel of rare earth elements (REEs) and other minor or technologically critical elements, including gallium (Ga), yttrium (Y), niobium (Nb), ruthenium (Ru), indium (In), lanthanum (La), cerium (Ce), praseodymium (Pr), neodymium (Nd), samarium (Sm), europium (Eu), gadolinium (Gd), terbium (Tb), dysprosium (Dy), holmium (Ho), erbium (Er), thulium (Tm), ytterbium (Yb), lutetium (Lu), tantalum (Ta), osmium (Os), platinum (Pt), and gold (Au). The inclusion of this extended set enables a robust assessment of both the essential mineral profile and contaminant exposure in the studied animals, while also addressing the emerging concern of REEs as diffuse pollutants derived from modern technological applications [19].

All elemental standards were obtained from CPA Chem (Stara Zagora, Bulgaria) as certified 100 mg/L stock solutions in 5% nitric acid (HNO_3_). Two distinct 10-point calibration series were employed to accommodate the broad diversity of elements: the first was based on a commercial multi-element solution that included the principal essential and toxic metals, and the second was a customized mixture prepared in-house using individual standards, specifically designed to cover REEs and other emerging elements not included in the commercial mix. Calibration curves extended from 0.005 to 20 ng/mL, and linearity was confirmed in all cases with regression coefficients (R^2^) exceeding 0.998.

Sample digestion was carried out using a microwave-assisted system (Milestone Ethos Up, Milestone Srl, Bologna, Italy). For each camel, two separate 250 mg aliquots of serum were processed using a mixture of 3.5 mL of ultrapure Milli-Q water and 1.25 mL of 65% sub-boiling nitric acid. The digestion program included four consecutive steps at increasing temperatures: 100 °C for five minutes, 150 °C for five minutes, 200 °C for eight minutes, and 200 °C for an additional seven minutes, all carried out at a constant power of 1800 W. After cooling, the digests were diluted to a final volume of 7.5 mL with ultrapure water. An internal standard solution containing scandium (Sc), germanium (Ge), rhodium (Rh), and iridium (Ir), each at 20 µg/mL, was added to every sample immediately prior to instrumental analysis to control for potential signal drift and matrix effects. Procedural blanks were prepared identically for every batch of samples.

Elemental quantification was conducted using an Agilent 7900 inductively coupled plasma mass spectrometer (ICP-MS; Agilent Technologies, Tokyo, Japan), equipped with a MicroMist concentric glass nebulizer, nickel cones, and an Ultra High Matrix Introduction (UHMI) system. To minimize spectral interferences, the Octopole Reaction System (ORS4) was operated in helium collision cell mode. Instrument tuning was performed daily using a standard optimization solution containing Cs, Co, Li, Mg, Tl, and Y. Sample introduction was performed using the Integrated Sample Introduction System (ISIS) in discrete mode. Data were acquired and processed with MassHunter v4.2 software (Agilent Technologies), and calibration-based quantification was applied across all elemental groups.

The analytical method was validated based on previously published protocols [20,21,22]. Recovery rates for essential, toxic, and emerging elements ranged between 87% and 128%. Precision was assessed through repeated analysis of spiked blank matrices at three concentrations (0.05, 0.5, and 5 ng/mL). Relative standard deviations (RSDs) were generally below 8% across all concentrations, although slightly higher variability (15–16%) was observed for elements such as Cu, Ni, Se, Sm, Fe, Ba, and Zn at the lowest concentration level. At higher concentrations, all RSDs were below 5%. Limits of quantification (LOQs) were determined from 20 replicates of procedural blanks, with each LOQ calculated as three times the standard deviation of the mean blank signal from procedural blanks.

### 2.3. Organic Contaminant Analysis

To characterize the chemical burden of dromedary camels from the Canary Islands in addition to elemental exposure, we applied a multiresidue analytical method capable of detecting and quantifying a broad spectrum of serum-borne organic pollutants. This method, developed and validated by our research group, enables the simultaneous determination of 353 compounds representing a wide range of environmental and veterinary contaminants [23]. The target analytes include organochlorine and organophosphorus pesticides, carbamates, pyrethroids, fungicides, herbicides, persistent organic pollutants (POPs) such as polychlorinated biphenyls (PCBs), polycyclic aromatic hydrocarbons (PAHs), brominated flame retardants (BDEs), rodenticides, veterinary pharmaceuticals, and selected industrial byproducts. The method was originally designed for biomonitoring in wildlife and livestock, and its robustness and scope make it particularly well suited to screening non-traditional livestock species such as camels.

Sample preparation followed a modified QuEChERS protocol adapted for low-volume biological matrices. One milliliter of serum was extracted with acetonitrile acidified with 2.5% formic acid to facilitate protein precipitation and analyte release. Salts, including magnesium sulfate and sodium acetate, were added as partitioning salts to facilitate phase separation and improve recovery. After sonication and orbital shaking, the mixture was centrifuged and filtered. To further reduce matrix effects, especially those associated with serum lipids and residual proteins, the supernatant was subjected to a freeze-out purification step involving two successive cycles of freezing at −24 °C followed by centrifugation. This simple yet effective clean-up procedure enables direct injection of the extract into both LC-MS/MS and GC-MS/MS systems without the need for additional solid-phase extraction steps or solvent exchange, thereby reducing variability and cost.

Instrumental analysis was performed using two complementary platforms to ensure comprehensive analyte coverage. LC-MS/MS determinations were carried out using a 1290 Infinity II system coupled to an Agilent 6460 Triple Quadrupole mass spectrometer equipped with a Jet Stream electrospray source. Chromatographic separation was achieved on a Poroshell 120 EC-C18 column using a binary gradient composed of aqueous ammonium acetate and methanol. The system operated under dynamic multiple reaction monitoring (dMRM) conditions, with compound detection in both positive and negative ionization modes, depending on polarity and functional group characteristics. GC-MS/MS analysis was conducted on an Agilent 7890B gas chromatograph coupled to a 7010 Triple Quadrupole mass detector. Separation was achieved using a backflush configuration with two HP-5MS columns, under electron impact ionization and timed MRM acquisition across 24 segments. Analytical conditions were optimized for sensitivity, reproducibility, and minimal carryover.

Quantification was based on matrix-matched calibration curves, prepared in triplicate over a range of 0.002 to 80 µg/kg, and validated through internal standards and fortified control samples. Quality control was ensured by including procedural blanks and a spiked matrix control every 20 samples. Identification relied on the detection of two MRM transitions with retention time matching (±0.1 min) and acceptable ion ratios (±30% of reference values). For chiral compounds, enantiomeric quantification was performed when required by residue definitions. Validation criteria—including limits of detection and quantification, linearity, recovery, and precision—were met for all 353 analytes, in line with current EU guidelines for the analysis of residues in food and biological matrices [24,25].

The full validation of the method, including detailed chromatographic and mass spectrometric conditions for each compound, has been published elsewhere [23,26,27] and is not reproduced here in full for reasons of brevity.

## 3. Results

Results are presented by analyte type, starting with inorganic elements (Section 3.1), followed by organic compounds (Section 3.2). Comparisons by age and reproductive status used the following definitions: calves < 12 months; adults ≥ 12 months; pregnancy based on farm records plus clinical assessment at sampling. Within each category, descriptive statistics are provided, with emphasis on differences according to sex, age, and pregnancy status.

### 3.1. Inorganic Elements

#### 3.1.1. Essential and Macroelements

The elemental composition of serum samples from dromedary camels (*Camelus dromedarius*) showed considerable interindividual variability, particularly among essential trace elements. Table 1 presents the mean, standard deviation, and median values of each analyte, along with statistical comparisons by sex, age group (adult vs. calf), and pregnancy status.

Among essential elements, Se, Fe, and Cu showed significant sex-related differences, with higher concentrations observed in females for Fe (*p* < 0.001) and in males for Se and Cu (*p* < 0.001 for both). Conversely, Mn concentrations were significantly higher in females (*p* = 0.004). Age also had a marked effect: calves exhibited significantly higher levels of Zn and Mn (*p* < 0.001), whereas adults had greater concentrations of Co and Se (*p* < 0.001). Pregnancy status was associated with decreased serum levels of Fe, Zn, and Se, all of which were lower in pregnant females compared with non-pregnant individuals (*p* < 0.05).

Regarding macroelements, serum concentrations of sodium (Na) and calcium (Ca) were slightly higher in males (*p* = 0.028 and *p* = 0.012, respectively), while age-related differences were more pronounced. Calves exhibited significantly higher concentrations of phosphorus (P), sulfur (S), and calcium (*p* < 0.001), whereas adults had higher levels of potassium (K). Pregnancy was associated with significant differences in magnesium (Mg), phosphorus, and potassium, with elevated Mg and K concentrations and reduced P levels observed in pregnant individuals (all *p* < 0.01).

#### 3.1.2. Toxic Elements and Potentially Toxic Elements

Serum concentrations of four classical toxic elements (As, Cd, Hg, and Pb) were generally low in the camel cohort. Most values were below the detection limit, with median concentrations of zero for all four elements. Descriptive and comparative statistics for these toxicants, as well as for a selection of potentially toxic elements, are presented in Table 2.

Hg levels showed a significant age-related difference, with higher concentrations in adults compared to calves (*p* = 0.011). Pb was significantly higher in pregnant females compared to non-pregnant ones (*p* = 0.001), although all median values remained at zero.

Among the potentially toxic elements, Sr and Ba were the most abundant, with mean concentrations of 172 µg/L and 40.1 µg/L, respectively. Both elements exhibited statistically significant differences by sex and age group. Sr concentrations were higher in males (*p* = 0.001) and in calves compared to adults (*p* < 0.001), while Ba levels were higher in males (*p* = 0.014), adults (*p* < 0.001), and non-pregnant females (*p* < 0.001).

Sb, Pt, and the total concentration of rare earth elements (REEs) were also detected at low concentrations, though they showed several significant differences. Sb was higher in pregnant females (*p* < 0.001), while Pt levels were associated with all three physiological variables: higher in males (*p* = 0.047), in calves (*p* = 0.002), and in pregnant females (*p* = 0.001). The aggregate REE concentration—calculated as the sum of individual lanthanides quantified—was used as a composite indicator due to their high collinearity and common factor structure observed in a preliminary principal component analysis. This variable was significantly higher in males (*p* = 0.025), in adults (*p* = 0.004), and in non-pregnant females (*p* = 0.036). In all cases, median values for Pt and REEs remained at zero.

### 3.2. Organic Contaminants

A total of 360 organic compounds were included in the multi-residue screening method applied to camel serum. These analytes comprised a wide array of chemical groups, including pesticides (herbicides, insecticides, fungicides), veterinary drugs, human pharmaceuticals, and persistent organic pollutants (POPs), such as polycyclic aromatic hydrocarbons (PAHs) and organochlorine pesticides.

Despite the comprehensive analytical scope, only 13 compounds (3.6%) were detected in at least one animal. Based on the individual-level data, 73 of the 114 camels (64.0%) showed detectable concentrations of at least one organic contaminant, while the remaining 41 animals (36.0%) had no detectable residues. This distribution suggests low yet non-negligible background exposure among the population.

Supplementary Table S1 provides a full list of the analytes included in the screening method, categorized by compound type and regulatory status within the European Union. Of the 13 compounds detected, at least four substances—including dieldrin, hexachlorobenzene, thiacloprid, and acephate—are no longer authorized for agricultural use in the EU, highlighting the potential relevance of legacy contaminants or banned compounds still circulating in trace amounts.

Table 3 summarizes the analytical results for the compounds detected, including detection frequency, mean ± standard deviation, median, and concentration range. The most frequently detected compound was the PAH acenaphthene, found in 63 camels (55.3%), followed by the fungicide procymidone (32.5%) and the PAH fluorene (12.3%). Several other compounds, including fungicides (thiabendazole, imazalil), insecticides (acephate, chlorantraniliprole, thiacloprid), pharmaceuticals (acetaminophen and penicillin G), and two POPs (hexachlorobenzene and dieldrin), were detected in fewer than 5% of the animals. For all detected compounds, concentration levels were consistently low, with most median values below 1 µg/L and minimal dispersion.

## 4. Discussion

The discussion follows the same structure as the results and is divided into inorganic (Section 4.1) and organic contaminants (Section 4.2). Each subsection interprets the main patterns observed in relation to camel physiology and environmental exposure.

### 4.1. Inorganic Elements

#### 4.1.1. Essential and Macroelements

The observed elemental profiles in camel serum reflect both physiological demands and potentially adaptive mechanisms to the semi-extensive management conditions under which these animals were raised. Higher concentrations of Se and Fe in males may reflect sex-related physiological variation; however, studies in livestock show inconsistent patterns, and these differences are often influenced more by species, diet, environment, and physiological state than by sex alone [28,29]. Cu levels also followed this pattern, which may relate to its role in mitochondrial oxidative processes and erythropoiesis [30,31].

Age-related differences were particularly evident. Calves presented elevated concentrations of Zn and Mn, two elements critical for growth, bone development, and enzymatic activity during early life [32,33]. Adult camels had significantly higher Co and Se levels. Although these differences were statistically significant, existing evidence suggests that hepatic storage and absorption of these elements in ruminants are more strongly influenced by dietary formulation and chemical form than by age per se [34]. These age-dependent differences underscore the importance of establishing reference intervals stratified by life stage, especially when interpreting these elements as biomarkers of nutritional or environmental status.

Pregnancy was associated with reduced serum concentrations of Fe, Zn, and Se, a finding consistent with the known fetal and placental demands for these micronutrients during gestation [35,36]. This observation suggests that even under stable feeding conditions, gestating females may require additional supplementation to maintain optimal trace element homeostasis, particularly for those elements involved in antioxidant defense and fetal development [37].

Among macroelements, P and S concentrations were higher in calves, aligning with their active skeletal growth and increased protein turnover [38,39]. K concentrations were slightly higher in adults, although the existing literature does not consistently support age-related differences in serum potassium levels among ruminants, suggesting that this variation may reflect individual or management-related factors rather than physiological maturity. Pregnancy-related increases in Mg and K, coupled with decreased phosphorus levels, may mirror the redistribution of electrolytes to meet fetal mineralization demands, as previously observed in pregnant heifers [36,40].

Macro-elements. The overall pattern of Ca, P, Mg, Na, and K was consistent with expectations for semi-extensive systems, with age-related differences attributable to growth demands and pregnancy-related shifts compatible with hemodilution and fetal mineral requirements. Although values clustered within physiologically acceptable ranges, these findings underscore the value of periodic nutritional review (diet and mineral supplementation) in late gestation and early growth to prevent subclinical imbalances that can affect productivity and welfare.

These results provide valuable baseline data for future nutritional and toxicological studies in camels. They also highlight the importance of accounting for physiological variability when interpreting trace and macroelement concentrations, particularly in food safety and animal health contexts.

In addition to providing a detailed chemical screening, this study offers observational reference intervals for a broad panel of essential and macroelements in clinically healthy camels managed under uniform feeding and housing conditions. These values, derived from a relatively large cohort, may serve as a baseline for future studies in camel nutrition, toxicology, and health monitoring. Particular attention is given to differences by sex, age, and reproductive status, with a focus on identifying potential needs for targeted mineral supplementation in physiologically vulnerable subgroups, such as pregnant females.

#### 4.1.2. Toxic and Potentially Toxic Elements

The serum concentrations of classical toxic elements (As, Cd, Hg, Pb) observed in this study were extremely low, with median values at or below the detection limit. These findings are consistent with the controlled management and limited environmental exposure of dromedary camels raised under semi-extensive systems in the Canary Islands. Given the matrix limitations, these very low serum concentrations should be interpreted as baseline values rather than as indicators of toxicological risk for animals or consumers. Risk assessment requires whole-blood or tissue data. Comparable low levels of As, Cd, and Pb have been reported in liver and muscle samples of camels raised in non-industrial regions of Iran [41] and in meat from Mauritania [9], supporting the use of camels as sentinel species in such contexts for background environmental contamination.

The slightly higher Hg levels observed in adults compared to calves may reflect cumulative exposure over time, as mercury is known to bioaccumulate in animal tissues, although serum is not its primary storage compartment. This matrix caveat is consistent with recent whole-blood biodistribution evidence in small ruminants under field conditions, further supporting caution in any cross-matrix comparison [42,43]. Similarly, the elevated Pb concentrations in pregnant females, despite their very low absolute values, could reflect mobilization from bone stores during gestation, a process documented in other mammalian species [44]. Hg, Pb, Cd, and As show a well-known tendency to partition into erythrocytes and certain tissues; serum is therefore not the optimal matrix for exposure assessment of these elements. Instead, serum data should be regarded strictly as preliminary baselines for surveillance purposes. They cannot be used to infer toxicological risk, particularly for elements with strong erythrocyte affinity, such as Pb or Hg.

Among the potentially toxic elements, the relatively elevated serum concentrations of Sr and Ba are noteworthy, although both elements are naturally occurring and can be present in water and forage without toxic consequences. Sr, in particular, shares metabolic pathways with Ca and may be physiologically regulated in bone metabolism [45]. The differences observed by sex and age group likely reflect physiological variation rather than environmental exposure. In the case of Pt and Sb—both of anthropogenic origin—the concentrations detected were extremely low, and their significance in animal health remains uncertain. However, elevated tissue levels of Sb have been documented in camels living near industrialized zones, suggesting environmental sources may lead to bioaccumulation under different conditions [46].

The rare earth elements (REEs), reported here as a composite variable, have received increasing attention as emerging environmental contaminants due to their rising industrial use and persistence. However, their baseline concentrations in domestic animals remain poorly characterized. In this study, REEs were rarely detected, and when present, concentrations were minimal. The grouping of individual lanthanides into a single metric, justified by their collinearity in principal component analysis, provides a useful approach to screen for their presence and facilitate future comparisons across studies. This strategy is supported by previous geochemical and environmental studies showing that PCA effectively captures the shared variance and grouping tendency of REEs due to their similar chemical properties [47].

Overall, this dataset offers an important contribution toward defining reference values for toxic and potentially toxic elements in dromedary camels under low-exposure conditions. While none of the substances analyzed appear to pose an immediate concern, continued monitoring is warranted, particularly as camels are increasingly integrated into food systems and may be exposed to novel anthropogenic pressures in the future.

### 4.2. Organic Contaminants

The results obtained from the organic contaminant screening confirm the limited environmental and dietary exposure of dromedary camels raised under semi-extensive management in the Canary Islands. Out of 360 compounds targeted in the analytical method, only 13 compounds (3.6%) were detected in any of the samples. Furthermore, although 64.0% of animals (73 out of 114) had measurable concentrations of at least one compound, these concentrations were consistently very low, typically well below 1 µg/L, with narrow interquartile ranges and low variability.

This low detection rate is consistent with prior studies on camel meat and tissues from regions with limited agrochemical input, including Mauritania and Iran, where concentrations of organic residues are generally minimal [11,15,16,48]. Camels managed under extensive or semi-extensive systems, particularly in arid zones, are often less exposed to intensive agricultural inputs than other livestock, which may explain the low prevalence of pesticide residues and veterinary drugs in their biological matrices.

Among the detected compounds, the most prevalent were acenaphthene and fluorene, both PAHs typically associated with incomplete combustion processes. The high frequency of acenaphthene (55.3%) may reflect diffuse background exposure via inhalation or ingestion of contaminated dust or soil particles, although its toxicological impact at these concentrations is likely negligible. Similar exposure routes for PAHs—especially acenaphthene—have been reported in agricultural areas subject to burning practices, where this compound has shown greater leachability than others and has been consistently detected at various soil depths [49].

The fungicide procymidone showed the highest concentration values among all detected compounds, with maximum levels exceeding 80 µg/L in some individuals. This finding is interpreted as evidence of recent exposure. Because MRLs are set for edible tissues and milk—not for serum—and kinetic bridging is unavailable, we refrain from any safety inference from these serum data. Imazalil and thiabendazole, also fungicides approved in the EU, were detected in isolated animals at low concentrations, consistent with background exposure under authorized agricultural practices [50].

The detection of acephate, chlorantraniliprole, and thiacloprid reflects the trace presence of insecticides, all of which are known to persist in environmental compartments. Acephate is a classical organophosphate, while chlorantraniliprole and thiacloprid belong to the neonicotinoid class, which has raised increasing concern due to its potential for sublethal and chronic effects on non-target species. The detection of thiacloprid, a neonicotinoid insecticide no longer authorized in the EU [50], is especially notable. Although concentrations in our samples were extremely low and likely of no toxicological concern, the presence of a banned compound highlights the importance of ongoing monitoring of legacy or illicit residues. To date, no studies have reported the prevalence or concentration range of thiacloprid residues in livestock tissues or fluids following its withdrawal from the European market, and existing data are limited to in vitro exposures or residues in plant matrices.

Among pharmaceuticals, acetaminophen (paracetamol) and penicillin G were detected in a small percentage of animals. While the presence of acetaminophen may be related to direct therapeutic use, an environmental origin cannot be ruled out, particularly given the widespread detection of this compound in aquatic ecosystems [51]. Penicillin G reached the highest concentration in the entire dataset (median 102.11 µg/L in two animals), suggesting recent administration, although no clinical records were available for confirmation.

The presence of organochlorine pesticides—specifically hexachlorobenzene and dieldrin—despite being banned for decades, is compatible with their known persistence and bioaccumulative properties. These compounds have often been detected in livestock from semi-arid environments, even in the absence of direct application, as they remain in soil and vegetation over long periods. Similar patterns have been reported in camels and other grazing species from North Africa and the Middle East [11,15].

An especially noteworthy finding was the complete absence of detectable residues of 4,4′-DDE and any PCB congeners in all serum samples analyzed. This finding is exceptional considering the historical ubiquity of these persistent organic pollutants in environmental matrices and animal tissues. Over the past two decades, environmental biomonitoring studies have consistently reported 4,4′-DDE and PCBs in livestock and wildlife, both in the Canary Islands [17,52] and in other regions [52,53,54]. To our knowledge, this is the first instance in which none of these compounds was detected in a sample set of this size. Although dromedary camels are strict herbivores and typically less exposed to these compounds than carnivores or omnivores, the absence of residues nonetheless represents a positive indicator of the low environmental burden in these production systems.

A limitation of this study is that all inorganic analyses were performed in serum, not whole blood. For toxic elements with high erythrocyte affinity (e.g., Pb, Hg), serum concentrations can underestimate systemic burdens relative to whole-blood biomonitoring. Accordingly, these data are intended as serum baselines, and cross-matrix comparisons should be made with caution.

Overall, the findings support the interpretation of dromedary camels as effective sentinels for baseline contaminant loads in low-intensity farming systems. The low detection rates, together with the scarcity of unauthorized substances and the consistently low concentration levels observed, reinforce their utility for environmental surveillance and food safety monitoring in marginal or semi-natural production contexts.

## 5. Conclusions

This study provides a comprehensive overview of physiological and toxicological serum profiles in dromedary camels (*Camelus dromedarius*) reared under semi-extensive conditions in the Canary Islands. Three categories of analytes were evaluated: essential elements, toxic and potentially toxic inorganic elements, and organic contaminants.

Essential elements, including both trace and macroelements, showed biologically relevant variations according to sex, age group, and pregnancy status. Selenium and copper were higher in males, while calves exhibited increased manganese and zinc levels. Pregnancy was associated with lower serum concentrations of iron, zinc, and selenium. These findings contribute to the establishment of preliminary reference intervals for healthy camels, stratified by physiological category, which may support veterinary nutritional assessment and herd health monitoring.

In contrast, toxic and potentially toxic elements such as arsenic, mercury, cadmium, and lead were present at extremely low or undetectable levels. Other elements like barium, strontium, and rare earth elements (REEs) were occasionally detected but showed no concentrations of toxicological concern. These data provide preliminary baselines for future monitoring but cannot be used to infer toxicological risk, particularly for elements with strong erythrocyte affinity such as Pb and Hg.

Organic contaminants were also rarely detected. Of the 360 compounds included in the analytical method, only 13 (3.6%) were detected in any animal, and concentrations were consistently low. The most common were acenaphthene, procymidone, and fluorene, with others such as acephate, penicillin G, and thiabendazole appearing sporadically. Notably, no residues of 4,4′-DDE or PCB congeners were found in any sample—a remarkable finding given the historical ubiquity of these compounds.

From a One Health perspective, the absence or trace-level detection of most targeted organic residues in serum suggests limited ongoing exposures under semi-extensive management, although regulatory assessment must rely on edible matrices. These serum baselines can inform environmental surveillance and risk communication with producers and authorities, and they provide a foundation for longitudinal monitoring of herds and areas.

Together, these results indicate that dromedary camels raised under low-input, semi-extensive systems in the Canary Islands exhibit low chemical exposure, making them a suitable sentinel species for environmental and food safety surveillance. The study also offers valuable reference data to guide future research and risk assessment in camel-based production systems.

**Practical take-home points for camel veterinarians:**

1. Use serum panels to obtain preliminary baselines at the herd level; for metals of toxicological concern (particularly Pb, Hg), individual risk assessment requires confirmation with whole-blood testing.
2. Review mineral supplementation in late gestation and young animals to prevent subclinical imbalances.
3. Maintain treatment records and withdrawal-time compliance; unexpected serum findings (e.g., antimicrobials) warrant record checks and follow-up.
4. Prioritize water/feed quality surveillance in semi-extensive systems as a preventive measure.

## Figures and Tables

**Table 1:** Descriptive and comparative statistics of essential and macroelements in camel serum by physiological status. Values for essential elements are expressed in µg/L and in mg/L for macroelements. Median values are included for descriptive purposes. Comparisons were made by sex, age group (adult vs. calf), and pregnancy status.

**(A) Essential Elements (µg/L)**									
**Element**	**Mean ± SD**	**Median**	**P25–P75 Range**	**Sex *p*-Value**	**Age *p*-Value**	**Pregnancy *p*-Value**	**Sex Difference**	**Age Difference**	**Pregnancy Difference**
Mn	3.01 ± 3.93	2.55	1.40–4.26	0.004	<0.001	0.822	♂ > ♀	Calf > Adult	n.s.
Fe	3031.8 ± 1684.2	2372.0	1810–4190	<0.001	0.034	0.008	♀ > ♂	Calf > Adult	No > Yes
Co	0.82 ± 0.54	0.73	0.52–1.00	0.205	<0.001	0.970	n.s.	Adult > Calf	n.s.
Zn	339.8 ± 89.5	322.0	281–392	0.870	<0.001	0.005	n.s.	Calf > Adult	No > Yes
Cu	626.2 ± 127.3	625.0	528–710	<0.001	0.375	0.354	♂ > ♀	n.s.	n.s.
Se	76.3 ± 58.2	60.8	40.0–95.3	<0.001	<0.001	0.018	♂ > ♀	Adult > Calf	No > Yes
Mo	6.87 ± 20.6	0.00	0.00–3.30	0.887	0.301	0.835	n.s.	n.s.	n.s.
(**B**) **Macroelements (mg/L)**									
**Element**	**Mean ± SD**	**Median**	**P25–P75 Range**	**Sex *p*-value**	**Age *p*-value**	**Pregnancy *p*-value**	**Sex Difference**	**Age Difference**	**Pregnancy Difference**
Na	3386 ± 208	3363	3245–3500	0.028	<0.001	0.053	♂ > ♀	Calf > Adult	n.s.
Mg	25.6 ± 2.24	25.6	23.9–27.1	0.550	0.284	<0.001	n.s.	n.s.	Yes > No
P	86.1 ± 22.1	77.5	69.3–98.5	0.078	<0.001	0.002	n.s.	Calf > Adult	No > Yes
S	878 ± 75.6	884	830–928	0.092	0.001	0.221	n.s.	Calf > Adult	n.s.
K	1025 ± 578	1229	730–1400	0.729	0.022	<0.001	n.s.	Adult > Calf	Yes > No
Ca	101 ± 10.0	100	93.0–108	0.012	<0.001	0.608	♂ > ♀	Calf > Adult	n.s.

Group definitions: calves < 12 months; adults ≥ 12 months. Pregnancy status: assigned from farm records and clinical assessment at sampling. Statistics: Mann–Whitney U or Kruskal–Wallis (non-parametric) as appropriate; two-tailed α = 0.05; <LOQ values imputed at LOQ/√2. Sex definition: ♀: females; ♂: males; >: higher than; n.s.: not significant differences between groups.

**Table 2:** Descriptive and comparative statistics of toxic and potentially toxic elements in camel serum. Values for toxic elements are expressed in µg/L unless otherwise indicated. Median and interquartile range (P25–P75) values are included for descriptive purposes. Comparisons were made by sex, age group (adult vs. calf), and pregnancy status.

**(A) Toxic Elements (µg/L)**									
**Element**	**Mean ± SD**	**Median**	**P25–P75 Range**	**Sex *p*-Value**	**Age *p*-Value**	**Pregnancy *p*-Value**	**Sex Difference**	**Age Difference**	**Pregnancy Difference**
As	0.1605 ± 0.8678	0.00	0.00–0.00	0.883	0.208	0.837	n.s.	n.s.	n.s.
Cd	0.0010 ± 0.0098	0.00	0.00–0.00	0.445	0.449	0.523	n.s.	n.s.	n.s.
Hg	0.0623 ± 0.1291	0.00	0.00–0.10	0.078	0.011	0.628	n.s.	Adult > Calf	n.s.
Pb	0.1646 ± 0.3905	0.00	0.00–0.00	0.301	0.679	0.001	n.s.	n.s.	Yes > No
(**B**) **Potentially Toxic Elements (µg/L)**									
**Element**	**Mean ± SD**	**Median**	**P25–P75 range**	**Sex *p*-value**	**Age *p*-value**	**Pregnancy *p*-value**	**Sex Difference**	**Age Difference**	**Pregnancy Difference**
Sr	172 ± 63	157	130–206	0.001	<0.001	0.594	♂ > ♀	Adult < Calf	n.s.
Ba	40.1 ± 15.2	37.4	30.7–48.0	0.014	<0.001	<0.001	♂ > ♀	Adult > Calf	No > Yes
Sb	3.53 ± 2.74	3.14	1.70–5.20	0.054	0.069	<0.001	♂ > ♀ (borderline)	n.s.	Yes > No
Pt	0.186 ± 0.280	0.00	0.00–0.30	0.047	0.002	0.001	♂ > ♀	Calf > Adult	Yes > No
Sum REEs	0.466 ± 0.970	0.00	0.00–0.15	0.025	0.004	0.036	♂ > ♀	Adult > Calf	No > Yes

Only elements detected in any of the samples are included in the table. Group definitions: calves < 12 months; adults ≥ 12 months. Pregnancy status: assigned from farm records and clinical assessment at sampling. Statistics: Mann–Whitney U or Kruskal–Wallis (non-parametric) as appropriate; two-tailed α = 0.05; <LOQ values imputed at LOQ/√2. Sex definition: ♀: females; ♂: males; >: higher than; n.s.: not significant differences between groups.

**Table 3:** Organic contaminants detected in camel serum.

Compound	Detected (n)	Detected (%)	Mean ± SD (µg/L)	Median (µg/L)	Range (µg/L)
Acenaphthene	63	55.3	0.59 ± 0.45	0.52	0.0–2.19
Procymidone	37	32.5	38.03 ± 21.37	33.17	3.43–86.20
Fluorene	14	12.3	0.59 ± 0.07	0.59	0.49–0.73
Acetaminophen (Paracetamol)	5	4.4	0.93 ± 0.25	0.84	0.68–1.25
Hexachlorobenzene	5	4.4	0.05 ± 0.01	0.05	0.04–0.05
Dieldrin	5	4.4	0.27 ± 0.22	0.32	0.03–0.56
Acephate	3	2.6	0.89 ± 0.34	0.72	0.68–1.28
Chlorantraniliprole	3	2.6	1.25 ± 1.64	0.33	0.28–3.14
Imazalil (Enilconazole)	2	1.8	0.29 ± 0.03	0.29	0.28–0.31
Thiabendazole	2	1.8	12.91 ± 17.26	12.91	0.71–25.12
Thiacloprid	2	1.8	0.17 ± 0.07	0.17	0.12–0.22
Penicillin G	2	1.8	102.11 ± 23.18	102.11	85.71–118.49
Pendimethalin	1	0.9	1.12	-	-

## Data Availability

Data are available from the corresponding author upon reasonable request.

## Supplementary Materials

The following supporting information can be downloaded at: https://www.mdpi.com/article/10.3390/vetsci12090829/s1. Table S1. Target list of the 360 organic compounds analyzed by LC–MS/MS or GC–MS/MS, including RT, transitions, and LOQs.
