# Supplementary material for: Comprehensive Profiling of Essential Elements and Organic and Inorganic Contaminants in Dromedary Camels from the Canary Islands: A Baseline for Nutritional and Environmental Assessment
Source: Vet Sci. 2025 Aug 29;12(9):829. doi: 10.3390/vetsci12090829 (PMC12474353; doi:10.3390/vetsci12090829)
Supplement: Supplementary file 1 [file vetsci-12-00829-s001.zip › vetsci-3789438-supplementary.pdf]

Supplementary Table S1. Complete List of Analytes in the Organic Contaminant Screening Method. This table summarizes the 360 compounds included in the multi-residue method, listing compound number, name, primary use category, and EU regulatory status.

No.	Compound	Use [a]	EU Status	No.	Compound	Use [a]	EU Status	No.	Compound	Use [a]	EU Status
1	2-Phenylphenol	F	Approved	2	4,4'-Dichlorobenzophenone (metabolite of dicofol)	Met	-	3	Abamectine	I, A, AH	Approved
4	Acenaphthene	POP	-	5	Acenaphtylene	POP	-	6	Acephate	I	Not approved
7	Acetaminophen (Paracetamol)	V, NSAID	Approved	8	Acetamiprid	I	Approved	9	Acrinathrin	I, A	Approved
10	Albendazole	V, AH	Approved	11	Aldicarb	I	Not approved	12	Aldicarb-sulfone	Met	-
13	Aldicarb-sulfoxide	Met	-	14	Aldrin	POP	-	15	Anthracene	POP	-
16	Atrazine	H	Not approved	17	Azinphos-methyl	I	Not approved	18	Azoxystrobin	F	Approved
19	BDE-28	POP	-	20	BDE-47	POP	-	21	BDE-85	POP	-
22	BDE-99	POP	-	23	BDE-100	POP	-	24	BDE-153	POP	-
25	BDE-154	POP	-	26	BDE-183	POP	-	27	Benalaxyl	F	Approved
28	Bendiocarb	I	Not approved	29	Bendiocarb metabolite (2, 2-dimethylbenzo-1, 3-dioxol-4-ol)	Met	-	30	Benfuracarb	I, AH	Not approved
31	Benzo[a]anthracene	POP	-	32	Benzo[a]pyrene	POP	-	33	Benzo[b]fluoranthene	POP	-
34	Benzo[ghi]perylene	POP	-	35	Benzo[k]fluoranthene	POP	-	36	Bifenthrin	I	Not approved
37	Bitertanol	F	Not approved	38	Boscalid (formerly nicobifen)	F	Approved	39	Brodifacoum	R	Not approved
40	Bromadiolone	R	Approved	41	Bromopropylate	A	Not approved	42	Bromuconazole (two isomers)	F	Approved
43	Bupirimate	F	Approved	44	Buprofezin	I	Approved	45	Cadusafos (ebufos)	I, AH	Not approved
46	Carbaryl	I	Not approved	47	Carbendazim (azole)	F	Not approved	48	Carbofuran	I, AH	Not approved
49	Carbofuran-3-hydroxy	Met	-	50	Carbosulfan	I, AH	Not approved	51	Cefuroxima axetil (two isomers)	V, MB	Not approved
52	Chloramphenicol	V, MB	Approved	53	Chlorantraniliprole	I	Approved	54	Chlorfenapyr	I, A	Not approved
55	Chlorfenvinphos	I	Not approved	56	Chlorobenzilate	A	Not approved	57	Chlorophacinone	R	Not approved

58	Chlorpropham	H	Not approved	59	Chlorpyrifos	I	Not approved	60	Chlorpyrifos methyl	I	Not approved
61	Chlorthal dimethyl	H	Not approved	62	Chrysene	POP	-	63	Clindamycin	V, MB	Approved
64	Clofentezine	A	Approved	65	Clothianidin	I	Not approved	66	Cloxacillin	V, MB	Approved
67	Cortiscosterone	V, GC	Not approved	68	Coumachlor	R	Not approved	69	Coumaphos	I, A	Not approved
70	Coumatetralyl	R	Not approved	71	Cyazofamid	F	Approved	72	Cyflufenamid	F	Approved
73	Cyfluthrin (sum of four isomers)	I	Not approved e	74	Cyhalothrin (lambda isomer)	I	Approved	75	Cymoxanil	F	Approved
76	Cypermethrin (sum of four isomers)	I	Approved f	77	Cyproconazole (two isomers)	F	Approved	78	Cyprodinil	F	Approved
79	Cyromazine	I, A	Not approved	80	Danofloxacin	V, MB	Approved	81	Dazomet	I, A, AH, F, H	Approved
82	Deltamethrin	I, A	Approved	83	Demeton-S-methyl	I, A	Not approved	84	Demeton-S-methyl-sulfone (Dioxydemeton)	I, A	Not approved
85	Dexamethasone	V, GC	Approved	86	Diazinon	I	Not approved	87	Dibenzo[a,h]anthracene	POP	-
88	Dichlorodiphenyldichloroethane (p,p' DDD)	POP	-	89	Dichlorodiphenyldichloroethylene (p,p' DDE)	POP	-	90	Dichlorodiphenyltrichloroethane (p,p' DDT)	POP	-
91	Diclofenac	V, NSAID	Approved	92	Dicloran	F, MB, WP	Not approved	93	Dicloxacillin	V, MB	Not approved
94	Dieldrin	POP	-	95	Diethathyl ethyl	H	Not approved	96	Diethofencarb	F, MB, WP	Approved
97	Difenacoum	R	Not approved	98	Difenoconazole	F, MB, WP	Approved	99	Difethialone	R	Not approved
100	Difloxacin	V, MB	Not approved	101	Diflubenzuron	I	Approved	102	Diflufenican	H	Approved
103	Dimethenamid-P (and its R-isomer)	H	Approved	104	Dimethoate	I	Not approved	105	Dimethomorph (two isomers)	F, MB, WP	Approved
106	Dimethylphenylsulfamide (DMSA, metabolite of dichlofluanid)	Met h	-	107	Diniconazole-M	F, MB, WP	Not approved	108	Dinocap	F, MB, WP	Not approved
109	Diphacinone	R	Not approved	110	Diphenylamine	PHP	Not approved	111	Dodine	F, MB, WP	Approved
112	Endosulfan alfa	POP	-	113	Endosulfan beta	POP	-	114	Endosulfan sulfate	POP	-

115	Endrin	POP	-	116	Enrofloxacin	V, MB	Approved	117	EPN	I, A	Not approved
118	Epoxiconazole	F	Approved	119	Eprinomectin	V, MB	Approved	120	Eritromicin	V, MB	Approved
121	Esfenvalerate	I	Approved	122	Ethion (diethion)	I, A	Not approved	123	Ethirimol	F, MB, WP	Not approved
124	Ethofumesate	H	Approved	125	Ethoprophos	I, AH	Not approved	126	Etofenprox	I, A	Approved
127	Etoxazole	A	Approved	128	Famoxadone	H	Approved	129	Fenamidone	F	Not approved
130	Fenamiphos	I, AH	Approved	131	Fenamiphos sulfone	Met	-	132	Fenamiphos sulfoxide	Met	-
133	Fenarimol	F, MB, WP	Not approved	134	Fenazaquin	A	Approved	135	Fenbendazole	V, AH	Approved
136	Fenbuconazole	F, V	Approved	137	Fenbutatin oxide	I, A	Not approved	138	Fenhexamid	F	Approved
139	Fenitrothion	I	Not approved	140	Fenoxycarb	I	Approved	141	Fenpropathrin	I, A	Not approved
142	Fenpropidin	F	Approved	143	Fenpropimorph	F	Not approved	144	Fenpyroximate	A	Approved
145	Fenthion	I, A	Not approved	146	Fenthion oxon	Met	-	147	Fenthion oxon sulfone	Met	-
148	Fenthion oxon sulfoxide	Met	-	149	Fenthion sulfone	Met	-	150	Fenthion sulfoxide	Met	-
151	Fenvalerate	I	Not approved	152	Fipronil	I, V	Not approved	153	Fipronil sulfide	Met	-
154	Flocoumafen	R	Not approved	155	Fluazinam	F	Approved	156	Flubendiamide	I	Approved
157	Flucythrinate (two isomers)	I, A	Not approved	158	Fludioxonil	F	Approved	159	Flufenoxuron	I, A	Not approved
160	Flumequine	V, MB	Approved	161	Flunixin	V, NSAID	Approved	162	Fluopyram	F	Approved
163	Fluoranthene	POP	-	164	Fluorene	POP	-	165	Fluquinconazole	F	Approved
166	Flusilazole	F, MB, WP	Not approved	167	Flutolanil	F, MB, WP	Approved	168	Flutriafol	F	Approved
169	Fluvalinate tau	I, A	Approved	170	Fonofos	I	Not approved	171	Formetanate	I, A	Approved
172	Fosthiazate	AH, V	Approved	173	Heptachlor	POP	-	174	Hexachlorobencene	POP	-
175	Hexachlorocyclohexane (alpha)	POP	-	176	Hexachlorocyclohexane (beta)	POP	-	177	Hexachlorocyclohexane (delta)	POP	-
178	Hexaclorocyclohexane (gamma, lindane)	POP	-	179	Hexaconazole (two isomers)	F, MB, WP	Not approved	180	Hexaflumuron	I	Not approved
181	Hexythiazox	A	Approved	182	Imazalil (enilconazole)	F, MB, WP, V	Approved	183	Imidacloprid	I	Approved
184	Indeno [1,2,3-cd] pyrene	POP	-	185	Indoxacarb	I	Approved	186	Iprodione	F, MB, WP	Not approved

187	Iprovalicarb	F	Approved	188	Isocarbophos	I	Not approved	189	Isofenphos methyl	I	Not approved
190	Isoprothiolane	F, MB, WP	Not approved	191	Ivermectin B1a	V, AH, A	Approved	192	Josamycin	V, MB	Not approved
193	Ketoprofen	V, NSAID	Approved	194	Kresoxim methyl	F	Approved	195	Leptophos	I	Not approved
196	Levamisole	V, AH	Approved	197	Lincomycin	V, MB	Approved	198	Linuron	F	Approved
199	Lufenuron	I	Not approved	200	Malaoxon	I	Not approved	201	Malathion	I	Not approved
202	Mandipropamid	F	Approved	203	Marbofloxacin	V, MB	Approved	204	Mebendazole	V, AH	Approved
205	Mefenamic acid	V, NSAID	Not approved	206	Mefenoxam (metalaxyl-M)	F	Approved	207	Meloxicam	V, NSAID	Approved
208	Mepanipyrim	F, MB, WP	Approved	209	Mepiquat	H	Approved	210	Metaflumizone	I	Approved
211	Metaldehyde	M	Approved	212	Metconazole	F	Approved	213	Methamidophos (two isomers)	I, A	Not approved
214	Methidathion	I, A	Not approved	215	Methiocarb	I, A, M	Not approved	216	Methiocarb-sulfoxide	Met	-
217	Methomyl	I, A, AH	Nor approved	218	Methomyl oxime	Met	-	219	Methoxyfenozide	I	Approved
220	Metoxychlor	POP	-	221	Metrafenone	F	Approved	222	Metronidazole	V, MB,	Approved
223	Mevinphos (phosdrin)	I, A	Not approved	224	Mirex	POP	-	225	Monocrotophos	I	Not approved
226	Myclobutanil	F, MB, WP	Approved	227	N-(2,4-dimethylphenyl)-N'-methylformamidine (DMPF, metabolite of amitraz)	Met g	-	228	N,N-dimethylformamidine (DMF, metabolite of amitraz)	Met g	-
229	N,N-Dimethyl-N'-p-tolylsulphamide (DMST, metabolite of tolyfluanid)	Met i	-	230	Nafcillin	V, MB	Not approved	231	Naphtalene	POP	-
232	Naproxen	V, NSAID	Not approved	233	Nitenpyram	I	Not approved	234	Novobiocin	V, MB	Not approved
235	Nuarimol	F, MB, WP	Approved	236	Ofurace	F, MB, WP	Approved	237	Omethoate	I, A	Not approved

238	Oxadixyl	F, MB, WP	Not approved	239	Oxamyl	I, A, AH	Approved	240	Oxfendazole	V, AH	Approved
241	Oxolinic acid	V, MB	Not approved	242	Oxydemeton methyl	I	Not approved	243	Oxyfluorfen	H	Approved
244	Paclobutrazol	H	Approved	245	Paraoxon methyl	I	Not approved	246	Parathion ethyl	I	Not approved
247	Parathion methyl	I	Not approved	248	PCB 28	POP	-	249	PCB 52	POP	-
250	PCB 77	POP	-	251	PCB 81	POP	-	252	PCB 101	POP	-
253	PCB 105	POP	-	254	PCB 114	POP	-	255	PCB 118	POP	-
256	PCB 123	POP	-	257	PCB 126	POP	-	258	PCB 138	POP	-
259	PCB 153	POP	-	260	PCB 156	POP	-	261	PCB 157	POP	-
262	PCB 167	POP	-	263	PCB 169	POP	-	264	PCB 180	POP	-
265	PCB 189	POP	-	266	Penconazole	F, MB, WP	Approved	267	Pencycuron	F, MB, WP	Approved
268	Pendimethalin	H	Approved	269	Penicillin G	V, MB	Not approved	270	Penicillin V	V, MB	Not approved
271	Permethrin	I, A	Not approved	272	Phenanthrene	POP	-	273	Phenylbutazone	V, NSAID	Approved
274	Phosalone	I, A	Not approved	275	Phosmet	I, A	Approved	276	Phosmet oxon	Met	-
277	Piperacillin	V, MB	Not approved	278	Pirimicarb	I	Approved	279	Pirimiphos ethyl	I, A	Not approved
280	Pirimiphos methyl	I, A	Approved	281	Prochloraz	F, MB, WP	Approved	282	Procymidone	F, MB, WP	Not approved
283	Profenofos	I, A	Not approved	284	Propamocarb	F	Approved	285	Propargite	A	Not approved
286	Propiconazole	A	Not approved	287	Propoxur	I	Not approved	288	Propyzamide (pronamide)	H	Approved
289	Proquinazid	F	Approved	290	Prothioconazol	F	Approved	291	Prothiophos	F	Not approved
292	Pymetrozine	I	Not approved	293	Pyraclostrobin	F	Approved	294	Pyrazophos	F, MB, WP	Not approved
295	Pyrene	POP	-	296	Pyridaben	I, A	Approved	297	Pyridaphenthion	I, A	Not approved
298	Pyrimethanil	F	Approved	299	Pyriproxifen	I	Approved	300	Quinalfos	I, A	Not approved
301	Quinoxyfen	F	Not approved	302	Rifampicin	V, MB	Not approved	303	Rotenone	I, R	Not approved
304	Roxithromycin	V, MB	Not approved	305	Sarafloxacin	V, MB	Not approved	306	Simazine	I	Not approved
307	Spinosad (two isomers)	I, V	Approved	308	Spiramycin (two isomers)	V, MB	Approved	309	Spirodiclofen	A	Approved

310	Spiromesifen	I	Approved	311	Spiroxamine	F	Approved	312	Strychnine	R	Not approved
313	Sulfacetamide	V, MB	Not approved	314	Sulfachloropiridacine	V, MB	Not approved	315	Sulfadiacine	V, MB	Approved
316	Sulfadimetoxine	V, MB	Approved	317	Sulfadoxine	V, MB	Approved	318	Sulfameracine	V, MB	Not approved
319	Sulfametacine	V, MB	Not approved	320	Sulfametizole	V, MB	Not approved	321	Sulfametoxazole	V, MB	Approved
322	Sulfametoxipiridacin e	V, MB	Not approved	323	Sulfamonomethoxine	V, MB	Not approved	324	Sulfapyridine	V, MB	Not approved
325	Sulfaquinoxaline	V, MB	Approved	326	Sulfatiazole	V, MB	Not approved	327	Sulfisoxazole	V, MB	Not approved
328	Tebuconazole	I, A	Approved	329	Tebufenocide	I	Approved	330	Tebufenpyrad	A	Approved
331	Teflubenzuron	I	Not approved	332	Tefluthrin	I	Approved	333	Telodrin (isobenzan)	I	Not approved
334	Terbufos	I, AH	Not approved	335	Terbuthylazine	H	Approved	336	Tetrachlorvinphos	I	Not approved
337	Tetraconazole	F, H	Approved	338	Tetradifon	A	Not approved	339	Tetramethrin	I	Not approved
340	Thiabendazole	AH, V	Approved	341	Thiacloprid	I	Approved	342	Thiamethoxam	I	Not approved
343	Thiophanate methyl	I	Approved	344	Tolclofos methyl	F, MB, WP	Approved	345	Tolfenamic acid	V, NSAID	Not approved
346	Triadimefon	F, MB, WP	Not approved	347	Triadimenol	F, MB, WP	Not approved	348	Triazophos (hostathion)	I, A	Not approved
349	Trichlorfon	I, AH, V	Not approved	350	Trifloxystrobin	F	Approved	351	Triflumizole	F	Approved
352	Triflumuron	I	Approved	353	Trifluralin	H	Not approved	354	Trimethoprim	V, MB	Approved
355	Triticonazole	F	Approved	356	Tylmicosin	V, MB	Approved	357	Tylosin	V, MB	Approved
358	Vinclozolin	F, MB, WP	Not approved	359	Warfarin	R	Not approved	360	Zoxamide	F	Approved

[a] POP – persistent organic pollutant; Non persistent pollutants: A – acaricide, MB – microbiocide, AH – anthelminthic, V – veterinary drugs, F – fungicide, H – herbicide, I – insecticide, R – plant growth regulator, WP – wood preservative, PHP – post-harvest preservative, M – Molluscicide, Met – metabolite, NSAID – nonsteroidal anti-inflamatory drug, GC – glucocorticoid
